# Digital Characteristics and Dissemination Indicators to Optimize Delivery of Internet-Supported Mindfulness-Based Interventions for People With a Chronic Condition: Systematic Review

**DOI:** 10.2196/mental.9645

**Published:** 2018-08-21

**Authors:** Lahiru Russell, Anna Ugalde, Donna Milne, David Austin, Patricia M Livingston

**Affiliations:** ^1^ School of Nursing and Midwifery Deakin University Geelong Australia; ^2^ Melanoma and Skin Services Peter MacCallum Cancer Center Melbourne Australia; ^3^ Department of Cancer Experiences Research Peter MacCallum Cancer Center Melbourne Australia; ^4^ School of Psychology Deakin University Geelong Australia

**Keywords:** mindfulness, internet, chronic condition

## Abstract

**Background:**

Internet-supported mindfulness-based interventions (MBIs) are increasingly being used to support people with a chronic condition. Characteristics of MBIs vary greatly in their mode of delivery, communication patterns, level of facilitator involvement, intervention period, and resource intensity, making it difficult to compare how individual digital features may optimize intervention adherence and outcomes.

**Objective:**

The aims of this review were to (1) provide a description of digital characteristics of internet-supported MBIs and examine how these relate to evidence for efficacy and adherence to the intervention and (2) gain insights into the type of information available to inform translation of internet-supported MBIs to applied settings.

**Methods:**

MEDLINE Complete, PsycINFO, and CINAHL databases were searched for studies assessing an MBI delivered or accessed via the internet and engaging participants in daily mindfulness-based activities such as mindfulness meditations and informal mindfulness practices. Only studies using a comparison group of alternative interventions (active compactor), usual care, or wait-list were included. Given the broad definition of chronic conditions, specific conditions were not included in the original search to maximize results. The search resulted in 958 articles, from which 11 articles describing 10 interventions met the inclusion criteria.

**Results:**

Internet-supported MBIs were more effective than usual care or wait-list groups, and self-guided interventions were as effective as facilitator-guided interventions. Findings were informed mainly by female participants. Adherence to interventions was inconsistently defined and prevented robust comparison between studies. Reporting of factors associated with intervention dissemination, such as population representativeness, program adoption and maintenance, and costs, was rare.

**Conclusions:**

More comprehensive descriptions of digital characteristics need to be reported to further our understanding of features that may influence engagement and behavior change and to improve the reproducibility of MBIs. Gender differences in determinants and patterns of health behavior should be taken into account at the intervention design stage to accommodate male and female preferences. Future research could compare MBIs with established evidence-based therapies to identify the population groups that would benefit most from internet-supported programs.

**Trial Registration:**

PROSPERO CRD42017078665; https://www.crd.york.ac.uk/prospero/display_record.php?RecordID=78665 (Archived by WebCite at http://www.webcitation.org/71ountJpu)

## Introduction

### Background

Over the past two decades, mindfulness has become a major focus of research in health care settings for people with chronic conditions [1]. In clinical research, the application of mindfulness focuses on cognitive and emotional regulation to help patients cope better with their conditions [2]. There is evidence to support the use of mindfulness-based interventions (MBIs) such as mindfulness-based stress reduction (MBSR) and mindfulness-based cognitive therapy (MBCT) for mental and physical symptoms in people with cancer, cardiovascular disease, chronic pain, depression, and anxiety disorders [1].

The internet has become a tool to disseminate MBIs to a larger number of people, bypassing barriers related to cost and time commitments for in-person therapy [3], the need for a trained therapist [4], and reluctance to engage in group interventions [5]. Recently, a meta-analysis and a systematic review assessed the impact of internet-supported MBIs on mental health [6] and people with chronic physical conditions [7]. The studies showed promising results for improving mental health outcomes and alleviating symptom burden. The meta-analysis was conducted among a diverse group of people (with or without a chronic illness) and reported beneficial small-to-moderate effects of the interventions on depression, anxiety, stress, well-being, and mindfulness [6]. Nevertheless, as highlighted by the authors, interventions varied in their modes of delivery (mobile phone app, website, or Web-based classroom), therapeutic approaches (mindfulness-based vs mindfulness-inspired interventions), and level of therapist involvement (self-guided vs therapist-guided) [6]. This makes it difficult to determine which aspects of the Web-based designs optimized intervention delivery and their associated behavior change. Another systematic review involving people with physical conditions showed a positive effect of the intervention compared with usual care on a variety of outcomes such as pain acceptance, coping measures, and depressive symptoms [7]; however, results were mixed when the interventions were compared with an active control group, such as cognitive behavioral therapy. The intervention delivery mode was broadly categorized into synchronous (ie, real-time delivery such as instant messaging, telephone, or videoconferencing) versus asynchronous (such as emails) and facilitated (therapist or moderator involvement) versus self-guided. Interventions can therefore vary across a wide range of digital features used for various purposes. For example, sending reminders and providing personalized feedback through emails are both asynchronous functions but may influence intervention engagement differently [8,9]. It is, therefore, important to examine the technology used in internet-supported MBIs to understand how digital functions optimize intervention delivery and outcomes.

The challenges of translating research findings into practice are well documented [10,11]. The lack of measures assessing generalizability and sustainability of interventions in trials is a critical factor hindering translation of findings [10]. Pragmatic frameworks used for program implementation and outcome evaluation can help bridge the gap between scientific knowledge and dissemination [12]. These frameworks tend to combine factors ascertaining the internal validity of a program, such as changes in outcomes of interest and attrition and adherence rates, with concepts relevant to external validity, such as representativeness of study population, availability and cost of resources, and organizational readiness [12], which may have particular relevance for Web-based mindfulness interventions. Hence, assessing the efficacy of internet-supported MBIs while collecting information relevant to its generalizability will provide important information on the potential impact on wider communities [13].

### Aims

The aims of this review were to (1) provide a description of digital characteristics of internet-supported MBIs and examine how these relate to evidence for efficacy and adherence to the intervention and (2) gain insights into the type of information available to inform translation of internet-supported MBIs to applied settings.

## Methods

### Review Process

This systematic review was conducted according to the Preferred Reporting Items for Systematic Reviews and Meta-analyses guidelines [14]. Due to the heterogeneity of the study designs, populations, and outcomes, a narrative synthesis of the results was conducted rather than a meta-analysis. The protocol was registered on PROSPERO database on 01/11/2017 with reference number CRD42017078665.

### Eligibility Criteria

The review focused on internet-delivered MBIs for people with a chronic condition. Inclusion criteria were structured according to the PICOS framework [15]. The PICOS acronym stands for patient (P); intervention (I); comparison, control, or comparator (C); outcome (O); and study type (S) and is described in more detail below.

#### Participants

Participants were aged 18 years or older and diagnosed with a chronic condition such as, but not limited to, heart disease, diabetes, cancer, respiratory disease, or mental illness (eg, depression).

#### Interventions

Interventions were MBIs that met the following two criteria: (1) delivered or accessed via the internet with at least 50% of interactions being technology-mediated and (2) engaging participants in daily mindfulness-based activities such as mindfulness meditations and informal mindfulness practices. Studies were excluded if they examined mindfulness as a component of another treatment such as acceptance and commitment therapy [16] and dialectical behavior therapy [17,18], as it was not possible to dissociate the effect of mindfulness from other components of the intervention.

#### Control Group

Control group could be comparison groups of alternative interventions (active comparator), usual care, or wait-list.

#### Outcome Measures

All outcome measures were considered.

#### Study Type

This review included original papers reporting on randomized, quasi-experimental, and feasibility or pilot studies comparing the efficacy of a Web-based MBI with a control group. Cross-sectional studies, case reports, review articles, dissertations, and commentaries were excluded from this review.

### Study Selection

Web-based psychoeducation studies were first evident in the literature in 2000 [19]. Hence, the literature search for this review was conducted between January 2000 and July 2017 across three Web-based databases (MEDLINE Complete, PsycINFO, and CINAHL) using the following search terms: Online (online OR internet OR “web-base” OR ehealth OR etherap* OR app* OR telehealth OR telemedicine), Mindfulness (mindful* OR MBSR OR MBCT OR “acceptance and commitment therapy” OR awareness OR meditat*), and Intervention (intervention* OR therap* OR group* OR treatment*). Identified papers and key review papers [6-8] were further examined for additional eligible studies.

Given the broad definition of chronic conditions (ie, long lasting with persistent effects [20], in which conditions may deteriorate, advance, fluctuate, or be characterized by remissions [21]), specific conditions were not included in the original search to maximize results.

Papers published in English that met the eligibility criteria were included in the review. Additionally, for any paper meeting the eligibility criteria, data were extracted from related papers describing different aspects of the same study (eg, methods paper and cost-effectiveness paper).

### Review Process

Titles of identified records were screened by one author (LR). Papers not meeting eligibility criteria were excluded at this stage, and abstracts of remaining papers were read by two authors (LR and DM). Full texts of abstracts meeting eligibility criteria were reviewed, and data were extracted by LR. In case of ambiguity, studies were discussed and agreed upon with coauthor AU.

### Data Extraction

#### Study Characteristics

A standardized data extraction form was developed to collect information about study design, assessment time points, primary outcomes measures, participant characteristics, intervention and control conditions, intervention adherence, study findings, and attrition rates.

Study findings were categorized into whether the intervention group had a statistically significant improvement (yes or no) on the primary outcomes at the postintervention assessment compared with the control group.

#### Digital Features of Internet-Supported Mindfulness-Based Interventions

Reporting on digital features was guided by a coding scheme developed by Webb and colleagues for Web-based interventions [22], and the features described in the studies have been included in this review. These features were divided into six main categories: (1) delivery mode, (2) navigational format, (3) automated communication, (4) additional material (eg, ebook, video, or audio files), (5) other features (eg, book or hard copy of intervention), and (6) level of facilitator involvement. On the basis of the information reported in the included studies, the delivery mode was further divided into Web-based, videoconference, and email-based. Navigational format was defined as tunneled (the intervention could only be experienced in a predetermined order, and modules, sessions, or Web pages could not be skipped) or flexible (the content of the intervention could be accessed according to the user’s preference, and modules, sessions, or Web pages could be skipped) [23]. Automated communication was divided into email reminders and follow-up messages to encourage participation. Facilitator involvement can vary substantially across interventions [8,24,25]; therefore, the level of involvement was summarized using criteria similar to those of another review [8]: interventions without any facilitator involvement were categorized as *none (self-guided)*; interventions where facilitators were only providing reminders, links to modules, encouragement, and answering logistical questions were categorized as having *low facilitator involvement*; *medium facilitator involvement* referred to the provision of feedback on homework for mastering mindfulness skills; and *high level of facilitator involvement* referred to the provision of intervention in person.

#### Internal and External Validity Indicators

Glasgow and colleagues developed a framework to evaluate the degree to which behavioral interventions reported on efficacy (internal validity) and generalizability to other settings and populations (external validity) [26]. More specifically, the framework focuses on the reporting of the following five dimensions: (1) the *reach* into the target population and representativeness of the study sample; (2) *efficacy or effectiveness* of the intervention on primary outcome(s) tested under either restricted or controlled or real-world conditions, quality of life, and avoidance of unintended or negative consequences; (3) *adoption* rates of organizations and staff that would use the intervention and the characteristics of those organizations and staff; (4) *implementation* of the intervention as intended; and (5) *maintenance* of the effects at the individual level and sustainability of the intervention at an organizational or delivery level (RE-AIM: reach, efficacy/effectiveness, adoption, implementation, maintenance). The RE-AIM framework has been used to review the literature in diverse health areas, such as physical activity during pregnancy [27] or among family caregivers [28], self-management programs for diabetes [29,30], and health literacy interventions [31].

The degree to which internal and external validity were reported was recorded using a 21-item validated data extraction tool capturing the five dimensions of the RE-AIM framework [32]. Each dimension comprises specific indicators that were rated as criteria met (*yes*) or not met (*no*) and had equal weight. Each indicator reported was given a score of 1 (see Multimedia Appendix 1).

##### Reach

The following information addressing the internal validity of each study was coded: methods used to identify the target population and its inclusion and exclusion criteria. The following indicators addressed external validity: the number of individuals who agreed to participate compared with the total number of eligible participants (participation rate) and the characteristics of participants compared with nonparticipants (representativeness).

##### Efficacy/Effectiveness

Efficacy studies investigate the effects of an intervention under highly controlled conditions with a homogenous patient population enrolled using strict inclusion and exclusion criteria [33]. Effectiveness or pragmatic studies examine interventions under conditions similar to real-world practice, such as routine clinical settings, with more heterogeneous patient populations. Effectiveness studies may also use a randomized controlled trial (RCT) design; however, the intervention is more often compared with usual care [33]. The efficacy/effectiveness dimension is also composed of indicators strongly associated with internal validity such as changes in primary outcomes and the proportion of participants lost at follow-up (attrition rate). Other indicators include the type of analysis conducted (ie, intention-to-treat or completer analysis) and measures of quality of life. We also examined if papers assessed changes in mindfulness scores and proposed a potential mechanism of action for mediation or moderation effects of these scores on the intervention [34]. For example, did the strength of the relationship between the intervention and the outcome vary according to participants’ mindfulness scores (moderation effect of mindfulness)? Or, does the intervention cause changes in mindfulness scores, which in turn impact the outcome measures (mediation effect)?

##### Adoption

This dimension assessed the extent to which an intervention is carried out at a staff and setting level. Papers were reviewed to identify the degree to which intervention settings were described (eg, primary care, outpatient clinics, and online forums). Additionally, methods to identify the staff who delivered the intervention and their level of expertise were also coded.

##### Implementation

The duration and frequency of the intervention, the extent to which the protocol was delivered as intended (adherence rate), and the cost of delivery were coded as indicators for the implementation dimension.

##### Maintenance

This dimension, also a measure of sustainability, was coded for indicators reporting on assessments 6 or more months after the completion of the intervention, the level of maintenance of the intervention, and the cost associated with this maintenance.

The quality of reporting on RE-AIM indicators was calculated for each study with a possible score ranging from 0 to 21. Following criteria from previous RE-AIM reviews [31,35], the reporting quality was categorized as high, moderate, or low for studies scoring 15-21, 8-14, or less than 8, respectively.

## Results

### Review Process

**Figure 1:**
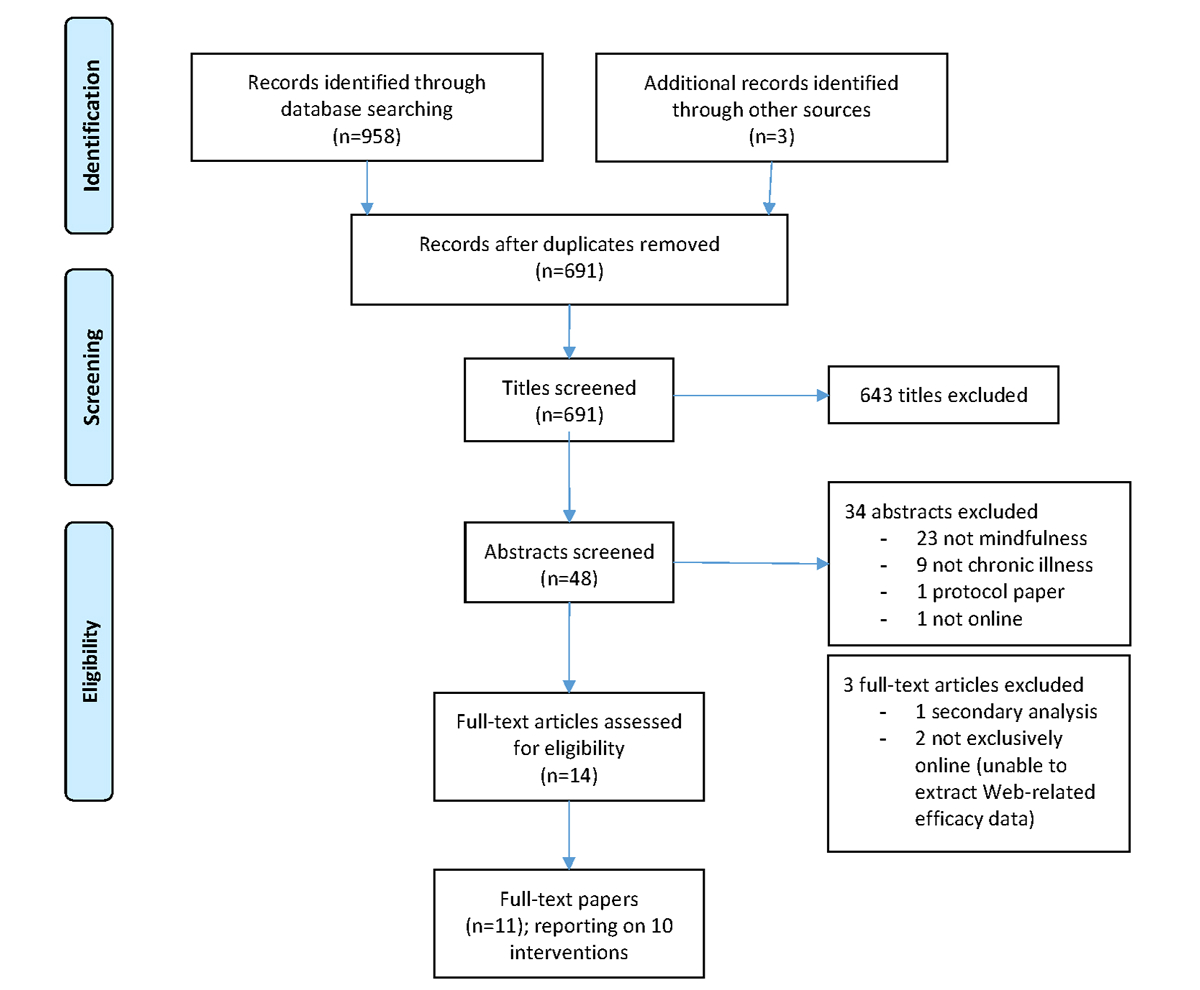
Flow diagram of the paper selection process.

A flow diagram of the selection process of the paper is provided in Figure 1.

The electronic database and external reference list searches produced 691 records after removal of duplicates. Title screening excluded 643 records leaving 48 abstracts that were examined, with 14 selected for full review. Two papers reported on findings from the same trial, one reported on the efficacy of the intervention to improve physical and psychological outcomes postintervention [36] and the other reported the 12-month follow-up assessment [37]. Both papers were included, but the methodology and findings were presented as one study. Another paper was a secondary analysis exploring the association of age, sex, and cancer stage on patient-reported outcomes postintervention [38]. This analysis did not include a comparative group and was therefore excluded from the review. Two other studies met the inclusion criteria, but their interventions combined two delivery modes (Web-based and telephone-based), and outcome data were not reported by delivery mode [39,40]. As effect size, attrition, and adherence rates for the Web-based group were not available, these latter studies were also excluded.

In total, 11 papers reporting 10 studies were included in the analysis.

### Study Characteristics

Table 1 provides a description of the study design, Table 2 provides a description of the participant characteristics, and Table 3 provides a description of the intervention and control conditions and a summary of intervention adherence, attrition rates, and overall outcome for each study.

Studies were published from 2008 and conducted in Sweden (n=3) [43,44,49], Canada (n=2) [41,45], the United States (n=2) [42,46], the Netherlands (n=1) [36], Ireland (n=1) [47], and Germany (n=1) [48]. Out of these, 8 studies were RCTs [36,42-45,47-49] and two were quasi-experimental designs [41,46]. Most studies involved female participants with an overall mean of 74.8%, ranging between 46.3% (150/324) [36] and 98% (77/98) [42]. The overall mean age of participants was 45 years (mean age range: 36-57.6 years). Three studies comprised active comparison groups, including the same MBI delivered in person [41], a Web-based behavioral activation condition [44], and a progressive muscle relaxation program [48]. Control conditions included attention control (n=1) [42], online discussion forum (n=2) [43,49], psychoeducational program (n=1) [47], wait-list (n=2) [41,45], or usual care (n=2) [36,46]. One study involved two comparison groups [41].

Chronic conditions examined in studies included chronic pain (n=3) [41,47,49], fibromyalgia (n=1) [42], heart disease (n=1) [36], cancer post-treatment (n=1) [45], anxiety disorder (n=1) [43], major depressive disorder (n=1) [44], residual depressive symptoms (n=1) [46], and psychosis (n=1) [48].

Primary outcome measures assessed pain or pain-related concepts (eg, pain interference, pain catastrophizing, pain-coping efficacy, and pain acceptance) in 4 studies [41,42,47,49]; depression in 3 studies [44,46,48]; distress in 2 studies [47,49]; and exercise capacity [36], anxiety [43], and mood disturbance [45] in one study each. In addition, 5 studies did not distinguish a primary outcome measure from their overall measures [41,42,47-49], which included constructs related to stress, affect, and quality of life. Study duration ranged from 6 weeks [42,48] to 12 months [36], with half of the studies including follow-up assessments [36,43,44,46,47] that ranged from 10 weeks [46] to 12 months [36]. Four studies assessed the participants’ mindfulness scores [45-47,49].

Duration of the interventions ranged from 6 [42,47,48] to 10 weeks [41], with half of the interventions conducted for 8 weeks [43-46,49]. Most studies demonstrated a benefit of the internet-supported MBI compared with a control condition [36,41-43,45,46,49]. However, in one study where the intervention was compared with two different conditions (wait-list and face-to-face MBI) [41], the intervention and the face-to-face MBI groups showed improvements in mental health–related quality of life, pain catastrophizing, and usual level of pain when compared with the wait-list group. No significant difference was observed between the intervention and face-to-face MBI groups. Similarly, 3 other studies [44,47,48] did not observe any difference between the intervention and control conditions, which were either active [44,48] or psychoeducational [47] conditions. In one study, a self-help manual about progressive muscle relaxation with audio files was emailed to the participants. The program showed to be as effective at improving depressive and obsessive-compulsive symptoms in people with psychosis as the MBI [48]. In another study, a pain management psychoeducational program and an MBI, comparable in delivery mode, structure, and time commitment, were both effective in improving pain-related outcomes and subjective well-being in people living with chronic pain [47]. In the third study, a subgroup analysis found that the intervention, a Web-based MBCT, worked better than the control condition among participants with milder depression, whereas the control condition, a behavioral activation program, had a greater effect on people with severe depression [44].

Adherence to the intervention was reported in 9 of the 10 studies, but the measure of adherence varied, from objective measures, such as the number of diaries or questionnaires completed, the number of Web-based *clicks*, or videoconference sessions attended, to participant self-report. In addition, adherence rate description was also varied, reported as either the mean proportion of sessions completed, the proportion of participants who completed all sessions or viewed all pages, or those who completed at least half of the program. Table 4 describes how intervention adherence was measured and defined in each study.

Attrition rates at postintervention follow-up ranged from 11% [43] to 62% [46] in the intervention groups, whereas attrition rates among the control conditions ranged between 0% [45,46] and 49% [41].

### Digital Features of the Interventions

Table 5 presents the main digital characteristics used in each intervention. Eight of the ten studies maintained the structure of in-person MBIs from which they were derived (ie, suggested a sequence of sessions or modules and recommendations for daily practice) [36,41-43,45-47,49]. However, the format was modified to match the way the internet-delivered interventions were typically applied, which resulted in briefer sessions and shorter meditation practices. The following section describes the digital features of each intervention based on their mode of delivery.

**Table 1:** Study design.

Reference	Study design	Time points	Primary outcome measures
Gardner-Nix et al, 2008 [41]	QE^a^	Pre, post	Physical and mental quality of life, pain catastrophizing, usual pain level, and pain-related suffering
Davis and Zautra, 2013 [42]	RCT^b^	Pre, daily for 6 weeks	Pain and pain coping efficacy, positive and negative affect, social activity engagement, loneliness, family stress, stress coping efficacy, and family enjoyment
Boettcher et al, 2014 [43]	RCT	Pre, post, 6-month follow-up	Anxiety
Ly et al, 2014 [44]	RCT	Pre, post, 6-month follow-up	Depression
Zernicke et al, 2014 [45]	RCT	Pre, post	Mood disturbance^c^
Dimidjian et al, 2014 [46]	QE	Pre, post, 10-weeks follow-up (FU1), 6-month follow-up (FU2)	Depression
Younge et al, 2015 [36]; Gotink et al, 2017 [37]	RCT	Pre, post, 12-month follow-up	Exercise capacity
Dowd et al, 2015 [47]	RCT	Pre, post, 6-month follow-up	Pain interference and distress
Moritz et al, 2015 [48]	RCT	Pre, post	Paranoia, obsessive-compulsive disorder, depression, and psychic experience scale
Henriksson et al, 2016 [49]	RCT	Pre, post	Pain intensity, pain acceptance, interference or suffering caused by pain, mindfulness, affective distress, life satisfaction, and life control

^a^QE: quasi-experimental.

^b^RCT: randomized controlled trial.

^c^Primary outcome was feasibility, but sample size was calculated with adequate power to reducing mood disturbance (secondary outcome).

**Table 2:** Participant characteristics.

Reference	Participant condition	Country	Females, n (%)	Age, mean (SD or range)
Gardner-Nix et al, 2008 [41]	Chronic pain	Canada	162 (75.3)	52 (32-79)
Davis and Zautra, 2013 [42]	Fibromyalgia	United States	77 (98)	46.14 (22-81)
Boettcher et al, 2014 [43]	Anxiety disorders	Sweden	65 (71)	38 (10.3)
Ly et al, 2014 [44]	Major depressive disorder	Sweden	57 (70)	36 (10.8)
Zernicke et al, 2014 [45]	Cancer recovery	Canada	45 (73)	57.6 (10.8)
Dimidjian et al, 2014 [46]	Residual depressive symptoms	United States	146 (73.0)	47.4 (11.43)
Younge et al, 2015 [36]; Gotink et al, 2017 [37]	Heart disease	The Netherlands	150 (46.3)	43.2 (27.5)
Dowd et al, 2015 [47]	Chronic pain	Ireland	112 (90.3)	44.5 (12.3)
Moritz et al, 2015 [48]	Psychosis	Germany	52 (58)	37.7 (9.7)
Henriksson et al, 2016 [49]	Chronic pain	Sweden	100 (93.5)	51 (9.3)

**Table 3:** Description of intervention and control conditions and summary of outcomes.

Reference	Control description	Intervention		Outcomes		
		Type	# of sessions, duration in weeks (intention)	Adherence to intervention, n (%)	Intervention group improvement over controls	Attrition (%)
Gardner-Nix et al, 2008 [41]	Active control on-site (C1) wait-list (C2)	MBCPM^a^	10 sessions per 10 weeks	NR^b^	Yes (except for physical quality of life and pain-related suffering)^c^	I^d^: 30; C1: 49; C2: 10
Davis and Zautra, 2013 [42]	Active control (health tips)	MSER^e^	12 modules per 6 weeks	Completed all modules, 19 (49)	Yes (except for pain and negative affect)	I: 15; C^f^: 5
Boettcher et al, 2014 [43]	Active control (discussion forum)	MBI^g^	16 hours per 8 weeks	All exercises completed on average, 46%	Yes	I: 11; FU^h^: 22; C: 4
Ly et al, 2014 [44]	Active control (behavioral activation)	MBCT^i^-inspired	8 weeks	Full adherence, 32 (78)	No, but after subgroup analysis: I>C for mild depression; C>I for severe depression	I: 12; FU: 17; C: 10; FU: 12.5
Zernicke et al, 2014 [45]	Wait-list	MBCR^j^	8 sessions per 8 weeks + 6 hours online silent retreat	Completed at least half the program (including retreat), 25 (83)	Yes	I: 27; C: 0
Dimidjian et al, 2014 [46]	Usual care	MBCT-inspired	8 sessions per 8 weeks	Completed all 8 sessions, 42 (42)	Yes	I: 62; FU1: 65; FU2: 73; C: 0
Younge et al, 2015 [36]; Gotink et al, 2017 [37]	Usual care	MBSR^k^-inspired	12 weeks	Completed at least half of the program, 115 (53.5)	Yes	I: 22; FU: 26; C: 16.5; FU: 22
Dowd et al, 2015 [47]	Psychoeducational	MBCT-inspired	12 sessions per 6 weeks	Viewed all sessions, 17 (74)	No	I: 55; FU: 63; C: 40; FU: 56
Moritz et al, 2015 [48]	Active control (progressive muscle relaxation)	MBI	6 weeks	Fully read the manual, 23 (61)	No	I: 26; C: 31
Henriksson et al, 2016 [49]	Active control (online forum)	MBSR-inspired	16 hours per 8 weeks	Completed full program, 18 (50)	Yes	I: 35; C: 21

^a^MBCPM: mindfulness-based chronic pain management.

^b^NR: not reported.

^c^Greater than wait-list, but not greater than onsite comparison group.

^d^I: intervention.

^e^MSER: mindful socioemotional regulation.

^f^C: control.

^g^MBI: mindfulness-based intervention.

^h^FU: follow-up.

^i^MBCT: mindfulness-based cognitive therapy.

^j^MBCR: mindfulness-based cancer recovery.

^k^MBSR: mindfulness-based stress reduction.

**Table 4:** Study definitions of intervention adherence and adherence rates.

Reference	Adherence defined as	Adherence rate
Gardner-Nix et al, 2008 [41]	Not reported	Not reported
Davis and Zautra, 2013 [42]	Number of diaries completed^a^	49% completed all modules
Boettcher et al, 2014 [43]	Number of *clicks*	46% of the mindfulness exercises completed
Dimidjian et al, 2014 [46]	Self-report	42% completed all sessions
Ly et al, 2014 [44]	At least one reflection emailed per week	78% completed all sessions
Zernicke et al, 2014 [45]	Number of videoconference sessions attended	83% completed at least half the program
Dowd et al, 2015 [47]	Self-report	74% viewed all sessions
Moritz et al, 2015 [48]	Self-report	61.5% fully read the manual
Younge et al, 2015 [36]	Number of assignments completed	53% completed at least half of the program
Henriksson et al, 2016 [49]	Self-report and verified by user-logged data	50% completed the full program

^a^Payment incentives for completing each diary.

**Table 5:** Digital features of internet-delivered mindfulness-based interventions.

Reference	Delivery mode	Navigational format	Automated communication	Additional material	Nondigital features	Level of facilitator involvement
Gardner-Nix et al, 2008 [41]	Videoconference	Tunneled	N/A^a^	CD	N/A	High
Davis and Zautra, 2013 [42]	Email-based	Tunneled	N/A	Animations and audios	N/A	Low
Boettcher et al, 2014 [43]	Web-based	Tunneled	Follow-up email	Video and audios	N/A	None (self-guided)
Dimidjian et al, 2014 [46]	Web-based	Flexible	N/A	Videos and audios	N/A	None (self-guided)
Ly et al, 2014 [44]	Web-based^b^	Flexible	N/A	Audios	N/A	Medium
Zernicke et al, 2014 [45]	Videoconference	Tunneled	N/A	Videos, audios, headsets, and webcam	Program manual	High
Dowd et al, 2015 [47]	Web-based	Flexible	Email reminders	Videos and audios	N/A	None (self-guided)
Moritz et al, 2015 [48]	Email-based	Flexible	N/A	Intervention manual (PDF) and audio files	N/A	None (self-guided)
Younge et al, 2015 [36]	Web-based	Flexible	Email reminders + follow-up text message	Videos and audios	Mindfulness book	None (self-guided)
Henriksson et al, 2016 [49]	Web-based	Flexible	Email reminders + follow-ups	Videos and audios	N/A	None (self-guided)

^a^N/A: not applicable.

^b^The intervention was delivered through a mobile app for iPhone owners or through a mobile phone−based app for other mobile phones.

### Web-Based Interventions

Web-based interventions were the most common mode of delivery with six interventions out of ten being accessible through websites [36,43,44,46,47,49]. One of these interventions was described as a mobile phone–based app [44], where the intervention was accessible through participants’ mobile phones. All six interventions offered meditation audio files and five offered a flexible navigational format [36,44,46,47,49]. Email reminders and follow-up messages were common features of these interventions, with four studies using automated email functionality [36,43,47,49] and one using therapist-initiated email [44]. Five of the 6 Web-based interventions were self-guided.

#### Email-Based Interventions

Two studies delivered the intervention via email, with one allowing for a flexible navigational format [48] and the other using a tunneled format [42]. In the former, participants were emailed a link to download a 15-page manual and four audio files providing instructions for meditation tasks. The study was self-guided, and there was no interaction with participants for the duration of the study period (6 weeks). In the latter study, participants were emailed one module of the intervention at a time, following completion of a diary. The material in each module was delivered via Adobe Presenter, which allowed visual presentation of texts and animated pictures that accompanied an audio recording of module content. Participants were also provided with audio recordings of mindfulness meditations and were encouraged to access the meditation daily.

#### Videoconference Intervention

Two studies offered MBI through a videoconferencing mode [41,45], which allowed for a synchronous delivery of the intervention and most closely resembled in-person formats.

Both programs consisted of weekly 2-hour sessions and the provision of meditation audio files. However, in one study [41], the intervention took place at the participant’s local hospital, whereas in the other study [45], participants accessed the intervention from their home through a Web-based educational platform that simulated a virtual classroom, where participants could see, hear, and interact in real time with other group members and the instructor. These two studies required a high level of facilitator involvement.

#### Internal and External Validity Indicators

Table 6 provides the proportion of internet-supported MBI studies reporting on RE-AIM dimensions and indicators.

##### Reach

Reach was the second most reported dimension at 66%. Studies consistently reported on the methodology for recruiting participants. Some studies recruited participants from known target populations, such as medical records [46], population registries [45,47], or outpatient clinics [36,41,49], and others employed a convenience sampling approach through the use of media outreach with Web-based and/or newspaper advertisements [42-45,48].

Inclusion and exclusion criteria were also regularly reported with only one study providing minimal description [41]. Two studies targeted mindfulness meditation–naïve participants and specifically excluded individuals with previous experience [43,45]. All studies reported on sample size, which ranged from 53 to 324 with a median of 99. Only 3 studies out of 10 provided information about participation rates [36,45,47], which were 31%, 36%, and 10%, respectively. None of the studies indicated the degree to which study samples were representative of a wider population; however, one study compared the baseline scores of the study sample on the mental component of a health-related quality of life questionnaire (SF-36v2) with the national population [41]. The mental health components comprised vitality, role emotional, social functioning, and mental health domains. These scores were 1.5 to 2 SDs below the average values of the national (US) population.

##### Efficacy/Effectiveness

With an overall of 75% indicators reported, efficacy or effectiveness was the most reported dimension across the studies. Changes in primary outcomes and attrition were described in all studies, but only half reported results of at least one follow-up [36,43,44,46,47], with 6-month follow-up being most common. Intention-to-treat analysis was used by majority of the studies (9 out of 10), one study reported on present-at-follow-up data [41], and one study reported on both intention-to-treat and completer analysis [36].

Most studies (n=7) reported on efficacy or effectiveness, with two [43,45] reporting on efficacy and five on effectiveness [36,41,42,48,49]. Six studies reported on quality of life or potential negative outcomes [36,41,43,44,47,49]. Five studies reported on interventions that improved participants’ quality of life [41,43,44,47,49], and one found no effect [36]. No negative outcomes were reported.

Only four studies examined changes in mindfulness as a result of their interventions. Of those, three studies reported an improvement in participants’ mindfulness scores [45,46,49], whereas one study reported a decrease [47].

##### Adoption

Adoption was the least reported dimension at 12%. Two studies described the staff who delivered the intervention—a trained research assistant [42], a study investigator, and a medical secretary [36]. Two other studies provided the level of expertise of the staff who delivered the intervention—a final year masters-level psychology student [44] and a clinician specialized in behavioral medicine with 15 years of experience in teaching MBSR [45]. Studies did not report on the identification of staff who delivered the intervention, inclusion and exclusion criteria of the delivery agent, or the adoption rate of the delivery agent.

Three studies described the intervention location—local hospitals [41], an outpatient clinic [36], and a cancer center [45]. Inclusion and exclusion criteria and adoption rate of settings were not reported.

##### Implementation

The mean proportion of reporting on implementation indicators was 63%. Intervention duration and frequency were reported by all studies. Nine out of ten studies reported on the extent to which the protocol was delivered as intended, but the cost of implementation was not reported in any study. Two studies offered monetary incentives to intervention participants by way of a gift voucher at enrolment [46] or a payment per returned diary [42].

##### Maintenance

Maintenance was the second least reported dimension at 17%. Half of the studies reported on outcome assessments at 6 months following the intervention.

Program-level maintenance and its associated costs were not reported in any study.

##### Overall Quality of Reporting on RE-AIM Indicators

The average reporting score was 9.4 out of 21, with scores ranging from 7 to 13. Two studies had low reporting quality, both with a score of 7 [41,48], and the other studies had moderate reporting quality with scores ranging from 8 [42,49] to 13 [36]. No study had a high reporting quality.

**Table 6:** Proportion of internet-delivered mindfulness-based intervention studies reporting on RE-AIM (reach, efficacy/effectiveness, adoption, implementation, maintenance) dimensions and indicators (N=10).

Indicator			Studies reporting, n (%)
**Reach**			
	1	Method to identify target population	10 (100)
	2	Inclusion criteria	10 (100)
	3	Exclusion criteria	9 (90)
	4	Participation rate	3 (30)
	5	Representativeness	1 (10)
	Average across reach indicators		6.6 (66)
**Efficacy/effectiveness**			
	6	Measures or results for at least one follow-up	5 (50)
	7	Intent-to-treat analysis	9 (90)
	8	Quality-of-life or potential negative outcomes	6 (60)
	9	Percent attrition	10 (100)
	Average across efficacy/effectiveness indicators		7.5 (75)
**Adoption**			
	10	Description of the intervention location	3 (30)
	11	Description of staff who delivered the intervention	2 (20)
	12	Method to identify staff who delivered the intervention (target delivery agent)	0 (0)
	13	Level of expertise of the delivery agent	2 (20)
	14	Inclusion and exclusion of the delivery agent or setting	0 (0)
	15	Adoption rate of the delivery agent or setting	0 (0)
	Average across adoption indicators		1.2 (12)
**Implementation**			
	16	Intervention duration and frequency	10 (100)
	17	Extent of the protocol delivered as intended	9 (90)
	18	Measures of the cost of implementation	0 (0)
	Average across implementation indicators		6.3 (63)
**Maintenance**			
	19	Assessed outcomes at 6 months or following post intervention	5 (50)
	20	Indicators of program-level maintenance	0 (0)
	21	Measures of the cost of maintenance	0 (0)
	Average across maintenance indicators		1.7 (17)

## Discussion

### Principal Findings

This review examined how digital features of internet-supported MBIs were related to the evidence of efficacy and intervention adherence and to which degree they informed capacity to translate into usual care using the RE-AIM framework. Since 2008, ten studies have examined the effects of an internet-delivered MBI on people with a chronic condition, with half of these studies published between 2014 and 2016. Findings indicated that internet-supported MBIs improved patient functioning for most outcome measures and were generally more effective than usual care or wait-list groups. Nevertheless, adherence to interventions was inconsistently and poorly defined and prevented robust comparison between studies. Self-guided interventions that allowed for flexible navigation of the program were as effective as facilitator-guided interventions, and more women with a chronic condition participated in an internet-supported MBI than men. This review also identified a number of reporting gaps within the RE-AIM framework, limiting the dissemination of internet-supported MBI research findings into practice.

### Intervention Efficacy

Overall, internet-delivered MBIs were more effective than usual care or wait-list groups but not more effective than an active condition. This was demonstrated in three of the studies included in this review [44,47,48]. When an active control group was similar to the intervention delivery mode, time commitment, and attention, both the groups showed improved patient outcomes. This is an observation common to RCTs in general, where the type of control condition is known to affect study outcomes [50]. The extent to which participants in the active condition group were provided with a credible treatment rationale may have influenced their experience of that condition by generating positive expectations for improvement [51]. An intervention aiming to improve emotion-related outcomes using a mindfulness-based program is likely to trigger positive outcome expectations among individuals struggling to cope with a chronic condition. A usual care or wait-list group that serves as an untreated comparator would in those circumstances be more likely to experience a negative expectancy bias, which may translate into poorer outcomes [52]. Interestingly, a Web-based behavioral activation program was found to be more effective for people with severe depression than its MBI equivalent. The MBI was, however, more effective for individuals with lower levels of depression than the behavioral activation group [44]. As depressed individuals generally tend to experience concentration difficulties, distractibility, and problems with effortful cognitive processes [53], the authors of the latter study suggested that interventions requiring substantial cognitive functioning, such as attention control practice in MBIs, may not suit severely depressed participants. These findings suggest that the use of an active comparator could help to discern particular individual characteristics more sensitive to a mindfulness-based program.

### Intervention Adherence

Adherence to the intervention was inconsistently defined across studies, which made comparison between studies difficult. This issue has previously been reported in other Web-based MBI reviews [6,7] and seems to endure across various types of behavioral Web-based research [54,55]. Adherence was broadly defined as the degree to which participants’ behavior followed the recommendations from those delivering the program [56]. However, a single measure of adherence was not always appropriate for complex interventions, such as MBIs, which combined multiple modalities including educational components, meditation exercises, and mindfulness practice, to which participants may differentially adhere [55,56]. For example, in a study assessing the efficacy of a mindfulness manual delivered by email, adherence was defined as the extent to which the manual was read by participants [48]. The mindfulness program described in the manual contained an introduction to the concept of mindfulness and an explanation of how mindfulness can be practiced. In addition, a CD was provided for meditation exercises. Although nearly two-thirds of the participants reported having fully read the manual, it is unclear to what extent formal and informal mindfulness exercises were practiced. It is also unclear whether participants correctly understood the concept of mindfulness. However, it is also important to note that behavior change prompted by internet-based interventions may not require sustained or in-depth engagement with the program, as some users may require only a short period of intense engagement to initiate a habit or learn a skill, whereas others may need longer periods and a more personalized approach [57]. This may partly explain the incongruity between low-adherence rates and improved outcome measures observed in this review (eg, [43,46]). Participants who deviated from the recommended MBI structure may have still benefited from some aspects of the intervention. Given the important role of each modality in MBIs (ie, educational, informal practice, and meditation exercises), reporting on a multimodal measurement of adherence would provide an understanding of which aspects of MBI impact effectiveness.

### Gender Disparity

Findings from this review were informed mainly by female participants (75%), which was slightly higher than those in in-person MBI studies where the average number of female participants was 71% [58]. Previous research showed that Web-based health-seeking behavior was reported to differ by gender, where women were more inclined to seek emotional and social support and affirmation of their health-related beliefs and men were interested mainly in health-related information [59]. Given the central focus of MBIs in health research is emotion regulation, these interventions may intuitively have a stronger appeal to women than to men. Gender differences in determinants and patterns of health behavior should be taken into account at the intervention design stage to accommodate male and female preferences.

### Digital Characteristics

The digital characteristics listed in this review reflected those reported by the individual studies and were not exhaustive [8,9]. The majority of interventions were self-guided, delivered through a Web or mobile app, and allowed for flexible navigation of the program. Other features such as presentation strategies, including page design principles, average amount of text on pages, and the presence of hyperlinks to other resources may not only further our understanding of features influencing engagement and behavior change but also improve the reproducibility of the intervention in other contexts [60]. This is particularly relevant for interventions with low to no facilitator involvement, as the impact of the intervention relies primarily on digital features. Features such as the provision of the same information through various channels (text, audio, and video) to accommodate individual learning preferences [61], automated reminders to meditate, invitations to provide reflection on personal practice, automated progression feedback, and a range of meditation files to choose from could optimize intervention effects and inform learning preferences of different cohorts.

### Internal and External Validity

This review used the RE-AIM framework to assess factors potentially hindering the translation of findings to clinical practice. Recognizing the RCT and quasi-experimental nature of the studies included, a focus on aspects related to internal validity was observed. Most indicators of reach, efficacy/effectiveness, and implementation were frequently reported across studies. However, within these domains, essential indicators of generalizability, sustainability, and cost-effectiveness were rarely or never reported. For example, within the Reach domain, data related to the representativeness of study population were seldom reported, as most studies failed to address denominators such as populations from which settings, health professionals, and patients were drawn. The absence of this information hinders an analysis of the potential representation of the sample with the general population [13]. It is recommended that future studies report beyond the characteristics of study participants by comparing them with those of people declining to participate. If the recruitment process occurs in health care settings where patients are individually introduced to the study, then characteristics of people declining could be collected either directly from them by explaining the importance of this information or from the organization’s database [13]. For online recruitment processes, existing databases such as population census data or national health surveys can be used to compare participants’ demographic characteristics to people in the same community [13].

Most studies reported whether the trial focused on efficacy or effectiveness and, in general, reported on indicators pertaining to these domains. The distinction between these two approaches lay in the objective of the study. Efficacy (or explanatory) studies aim to investigate, under strictly controlled conditions, the difference between two treatments, whereas effectiveness (or pragmatic) studies investigate how an intervention fares in *real world* settings [62]. Despite this theoretical difference, in behavioral research, efficacy and effectiveness studies are generally conducted in *real world* settings such as university teaching hospitals or community health clinics, involving actual patients with real health problems being treated in real health care services [52]. The type of real-world setting needs to be described to allow adequate interpretation of study outcomes and inform generalizability. In this regard, intervention location selection, description, and adoption rates were rarely reported indicators. In addition, although half the studies in this review reported on follow-up outcome assessments at 6 months or more, possible program adaptation and maintenance requirements were never discussed. Reporting on factors influencing intervention adoption and maintenance will help inform resource allocation, potential for program dissemination, and replication of interventions in other settings. Furthermore, none of the studies reported on aspects related to cost other than for participatory incentives [42,46]. Dissemination plans need to be informed by cost incurred at both organizational and individual levels. Understanding cost incurred by recruitment (eg, staff qualifications needed to recruit participants), technology, and program adaptation and maintenance (eg, fixing technical problems) will help organizations adequately evaluate dissemination opportunities. Furthermore, knowing about program data usage and the type of service plans and digital devices best suited for the program will inform future cost to participants, which will have an impact on reach and effectiveness of the intervention [60]. Hence, future RCTs need to report resources needed to conduct the study, as insight into financial consequences will have practical implications for dissemination.

Of note was that less than half of the studies used a mindfulness measure. This is, however, similar to in-person MBI research where mindfulness outcomes were assessed in only 45% of the studies [63]. In this review, studies did not propose a clear potential mechanism of action for mindfulness [34]. The extent to which mindfulness influences intervention outcomes remains unclear. To understand the mechanistic role of MBIs, it is suggested that studies consistently use mindfulness measures and report related outcomes within a proposed theoretical framework [64].

### Limitations

This review has several limitations that need to be considered when interpreting the results. First, data extraction was conducted by a single reviewer, which may have introduced some assessment bias. Second, digital characteristics described in this review were limited by the type of studies and populations included. Other study designs, such as pre-post studies, among, for example, a general population may describe additional features. Third, this review focused on the quality of reporting across the RE-AIM framework, which is different from the usual efficacy-based reviews that have a greater focus on the internal quality of the studies by performing risk of bias assessments [65]. However, two recent reviews of Web-based MBIs that focused on efficacy found that the quality of most studies was satisfactory and the interventions had a positive effect size on patient-reported outcomes [6,7]. These reviews both completed risk of bias assessments.

### Conclusions

Findings from this review suggest that self-guided MBIs and those with minimal facilitator involvement can help alleviate the psychological burden associated with chronic disease. Future research is recommended to compare these types of interventions with other more established evidence-based therapies to identify the population groups that would benefit most from internet-supported MBIs.

## Appendix

Multimedia Appendix 1 RE-AIM (reach, efficacy/effectiveness, adoption, implementation, maintenance) scoring instrument.

## Abbreviations

C: control group
FU: follow-up
I: intervention
MBI: mindfulness-based intervention
MBCPM: mindfulness-based chronic pain management
MBCT: mindfulness-based cognitive therapy
MBCR: mindfulness-based cancer recovery
MBSR: mindfulness-based stress reduction
MSER: mindful socioemotional regulation
NR: not reported
PICOS: intervention, comparison, control, or comparator, outcome, and study type
QE: quasi-experimental
RCT: randomized controlled trial
RE-AIM: reach, efficacy/effectiveness, adoption, implementation, maintenance

## References

[1] Gotink RA, Chu P, Busschbach JJ, Benson H, Fricchione GL, Hunink MG (2015). Standardised mindfulness-based interventions in healthcare: an overview of systematic reviews and meta-analyses of RCTs. PLoS One.

[2] Chambers R, Gullone E, Allen NB (2009). Mindful emotion regulation: an integrative review. Clin Psychol Rev.

[3] Boggs JM, Beck A, Felder JN, Dimidjian S, Metcalf CA, Segal ZV (2014). Web-based intervention in mindfulness meditation for reducing residual depressive symptoms and relapse prophylaxis: a qualitative study. J Med Internet Res.

[4] Waelde LC, Thompson JM, Robinson A, Iwanicki S (2016). Trauma therapists' clinical applications, training, and personal practice of mindfulness and meditation. Mindfulness (N Y).

[5] Wahbeh H, Svalina MN, Oken BS (2014). Group, one-on-one, or internet? Preferences for mindfulness meditation delivery format and their predictors. Open Med J.

[6] Spijkerman MP, Pots WT, Bohlmeijer ET (2016). Effectiveness of online mindfulness-based interventions in improving mental health: a review and meta-analysis of randomised controlled trials. Clin Psychol Rev.

[7] Toivonen KI, Zernicke K, Carlson LE (2017). Web-based mindfulness interventions for people with physical health conditions: systematic review. J Med Internet Res.

[8] Zhao D, Lustria ML, Hendrickse J (2017). Systematic review of the information and communication technology features of web- and mobile-based psychoeducational interventions for depression. Patient Educ Couns.

[9] Barak A, Klein B, Proudfoot JG (2009). Defining internet-supported therapeutic interventions. Ann Behav Med.

[10] Glasgow RE, Lichtenstein E, Marcus AC (2003). Why don't we see more translation of health promotion research to practice? Rethinking the efficacy-to-effectiveness transition. Am J Public Health.

[11] Riley WT, Glasgow RE, Etheredge L, Abernethy AP (2013). Rapid, responsive, relevant (R3) research: a call for a rapid learning health research enterprise. Clin Transl Med.

[12] Gaglio B, Phillips SM, Heurtin-Roberts S, Sanchez MA, Glasgow RE (2014). How pragmatic is it? Lessons learned using PRECIS and RE-AIM for determining pragmatic characteristics of research. Implement Sci.

[13] Glasgow RE (2007). eHealth evaluation and dissemination research. Am J Prev Med.

[14] Moher D, Liberati A, Tetzlaff J, Altman DG, PRISMA Group (2009). Preferred reporting items for systematic reviews and meta-analyses: the PRISMA statement. PLoS Med.

[15] Liberati A, Altman DG, Tetzlaff J, Mulrow C, Gøtzsche PC, Ioannidis JP, Clarke M, Devereaux PJ, Kleijnen J, Moher D (2009). The PRISMA statement for reporting systematic reviews and meta-analyses of studies that evaluate health care interventions: explanation and elaboration. PLoS Med.

[16] Hayes SC, Luoma JB, Bond FW, Masuda A, Lillis J (2006). Acceptance and commitment therapy: model, processes and outcomes. Behav Res Ther.

[17] Linehan MM (1993). Cognitive-Behavioral Treatment of Borderline Personality Disorder.

[18] Linehan MM (1993). Skills Training Manual for Treating Borderline Personality Disorder.

[19] Barak A, Hen L, Boniel-Nissim M, Shapira N (2008). A comprehensive review and a meta-analysis of the effectiveness of internet-based psychotherapeutic interventions. J Technol Hum Serv.

[20] Aihw.

[21] Helgeson VS, Zajdel M (2017). Adjusting to chronic health conditions. Annu Rev Psychol.

[22] Webb TL, Joseph J, Yardley L, Michie S (2010). Using the internet to promote health behavior change: a systematic review and meta-analysis of the impact of theoretical basis, use of behavior change techniques, and mode of delivery on efficacy. J Med Internet Res.

[23] Crutzen R, Cyr D, de Vries NK (2012). The role of user control in adherence to and knowledge gained from a website: randomized comparison between a tunneled version and a freedom-of-choice version. J Med Internet Res.

[24] Morrison LG, Yardley L, Powell J, Michie S (2012). What design features are used in effective e-health interventions? A review using techniques from critical interpretive synthesis. Telemed J E Health.

[25] Ugalde A, Haynes K, Boltong A, White V, Krishnasamy M, Schofield P, Aranda S, Livingston P (2017). Self-guided interventions for managing psychological distress in people with cancer - a systematic review. Patient Educ Couns.

[26] Glasgow RE, Vogt TM, Boles SM (1999). Evaluating the public health impact of health promotion interventions: the RE-AIM framework. Am J Public Health.

[27] Craike M, Hill B, Gaskin CJ, Skouteris H (2017). Interventions to improve physical activity during pregnancy: a systematic review on issues of internal and external validity using the RE-AIM framework. BJOG.

[28] Cuthbert CA, King-Shier K, Ruether D, Tapp DM, Culos-Reed SN (2017). What is the state of the science on physical activity interventions for family caregivers? A systematic review and RE-AIM evaluation. J Phys Act Health.

[29] Matthews L, Kirk A, Macmillan F, Mutrie N (2014). Can physical activity interventions for adults with type 2 diabetes be translated into practice settings? A systematic review using the RE-AIM framework. Transl Behav Med.

[30] Eakin EG, Bull SS, Glasgow RE, Mason M (2002). Reaching those most in need: a review of diabetes self-management interventions in disadvantaged populations. Diabetes Metab Res Rev.

[31] Allen K, Zoellner J, Motley M, Estabrooks PA (2011). Understanding the internal and external validity of health literacy interventions: a systematic literature review using the RE-AIM framework. J Health Commun.

[32] Glasgow RE, McKay HG, Piette JD, Reynolds KD (2001). The RE-AIM framework for evaluating interventions: what can it tell us about approaches to chronic illness management?. Patient Educ Couns.

[33] Singal AG, Higgins PD, Waljee AK (2014). A primer on effectiveness and efficacy trials. Clin Transl Gastroenterol.

[34] Edwards JR, Lambert LS (2007). Methods for integrating moderation and mediation: a general analytical framework using moderated path analysis. Psychol Methods.

[35] Blackman KC, Zoellner J, Berrey LM, Alexander R, Fanning J, Hill JL, Estabrooks PA (2013). Assessing the internal and external validity of mobile health physical activity promotion interventions: a systematic literature review using the RE-AIM framework. J Med Internet Res.

[36] Younge JO, Wery MF, Gotink RA, Utens EM, Michels M, Rizopoulos D, van Rossum EF, Hunink MG, Roos-Hesselink JW (2015). Web-Based mindfulness intervention in heart disease: a randomized controlled trial. PLoS One.

[37] Gotink RA, Younge JO, Wery MF, Utens EM, Michels M, Rizopoulos D, van Rossum LF, Roos-Hesselink JW, Hunink MM (2017). Online mindfulness as a promising method to improve exercise capacity in heart disease: 12-month follow-up of a randomized controlled trial. PLoS One.

[38] Zernicke KA, Campbell TS, Speca M, Ruff KM, Flowers S, Tamagawa R, Carlson LE (2016). The eCALM trial: eTherapy for cancer applying mindfulness. Exploratory analyses of the associations between online mindfulness-based cancer recovery participation and changes in mood, stress symptoms, mindfulness, posttraumatic growth, and spirituality. Mindfulness (N Y).

[39] Thompson NJ, Patel AH, Selwa LM, Stoll SC, Begley CE, Johnson EK, Fraser RT (2015). Expanding the efficacy of project UPLIFT: distance delivery of mindfulness-based depression prevention to people with epilepsy. J Consult Clin Psychol.

[40] Thompson NJ, Walker ER, Obolensky N, Winning A, Barmon C, Diiorio C, Compton MT (2010). Distance delivery of mindfulness-based cognitive therapy for depression: project UPLIFT. Epilepsy Behav.

[41] Gardner-Nix J, Backman S, Barbati J, Grummitt J (2008). Evaluating distance education of a mindfulness-based meditation programme for chronic pain management. J Telemed Telecare.

[42] Davis MC, Zautra AJ (2013). An online mindfulness intervention targeting socioemotional regulation in fibromyalgia: results of a randomized controlled trial. Ann Behav Med.

[43] Boettcher J, Aström V, Påhlsson D, Schenström O, Andersson G, Carlbring P (2014). Internet-based mindfulness treatment for anxiety disorders: a randomized controlled trial. Behav Ther.

[44] Ly KH, Trüschel A, Jarl L, Magnusson S, Windahl T, Johansson R, Carlbring P, Andersson G (2014). Behavioural activation versus mindfulness-based guided self-help treatment administered through a smartphone application: a randomised controlled trial. BMJ Open.

[45] Zernicke KA, Campbell TS, Speca M, McCabe-Ruff K, Flowers S, Carlson LE (2014). A randomized wait-list controlled trial of feasibility and efficacy of an online mindfulness-based cancer recovery program: the eTherapy for cancer applying mindfulness trial. Psychosom Med.

[46] Dimidjian S, Beck A, Felder JN, Boggs JM, Gallop R, Segal ZV (2014). Web-based mindfulness-based cognitive therapy for reducing residual depressive symptoms: an open trial and quasi-experimental comparison to propensity score matched controls. Behav Res Ther.

[47] Dowd H, Hogan MJ, McGuire BE, Davis MC, Sarma KM, Fish RA, Zautra AJ (2015). Comparison of an online mindfulness-based cognitive therapy intervention with online pain management psychoeducation: a randomized controlled study. Clin J Pain.

[48] Moritz S, Cludius B, Hottenrott B, Schneider BC, Saathoff K, Kuelz AK, Gallinat J (2015). Mindfulness and relaxation treatment reduce depressive symptoms in individuals with psychosis. Eur Psychiatry.

[49] Henriksson J, Wasara E, Rönnlund M (2016). Effects of eight-week-web-based mindfulness training on pain intensity, pain acceptance, and life satisfaction in individuals with chronic pain. Psychol Rep.

[50] Mohr DC, Spring B, Freedland KE, Beckner V, Arean P, Hollon SD, Ockene J, Kaplan R (2009). The selection and design of control conditions for randomized controlled trials of psychological interventions. Psychother Psychosom.

[51] Schwartz CE, Chesney MA, Irvine MJ, Keefe FJ (1997). The control group dilemma in clinical research: applications for psychosocial and behavioral medicine trials. Psychosom Med.

[52] Freedland KE, Mohr DC, Davidson KW, Schwartz JE (2011). Usual and unusual care: existing practice control groups in randomized controlled trials of behavioral interventions. Psychosom Med.

[53] Gotlib IH, Joormann J (2010). Cognition and depression: current status and future directions. Annu Rev Clin Psychol.

[54] Schubart JR, Stuckey HL, Ganeshamoorthy A, Sciamanna CN (2011). Chronic health conditions and internet behavioral interventions: a review of factors to enhance user engagement. Comput Inform Nurs.

[55] Ryan C, Bergin M, Wells JS (2018). Theoretical perspectives of adherence to web-based interventions: a scoping review. Int J Behav Med.

[56] Vitolins MZ, Rand CS, Rapp SR, Ribisl PM, Sevick MA (2000). Measuring adherence to behavioral and medical interventions. Control Clin Trials.

[57] Michie S, Yardley L, West R, Patrick K, Greaves F (2017). Developing and evaluating digital interventions to promote behavior change in health and health care: recommendations resulting from an international workshop. J Med Internet Res.

[58] Bodenlos JS, Strang K, Gray-Bauer R, Faherty A, Ashdown BK (2017). Male representation in randomized clinical trials of mindfulness-based therapies. Mindfulness.

[59] Baumann E, Czerwinski F, Reifegerste D (2017). Gender-specific determinants and patterns of online health information seeking: results from a representative German health survey. J Med Internet Res.

[60] Eysenbach G, CONSORT-EHEALTH Group (2011). CONSORT-EHEALTH: improving and standardizing evaluation reports of Web-based and mobile health interventions. J Med Internet Res.

[61] Russell SS (2006). An overview of adult-learning processes. Urol Nurs.

[62] Kowalski CJ (2010). Pragmatic problems with clinical equipoise. Perspect Biol Med.

[63] Khoury B, Lecomte T, Fortin G, Masse M, Therien P, Bouchard V, Chapleau MA, Paquin K, Hofmann SG (2013). Mindfulness-based therapy: a comprehensive meta-analysis. Clin Psychol Rev.

[64] Davidson RJ, Kaszniak AW (2015). Conceptual and methodological issues in research on mindfulness and meditation. Am Psychol.

[65] Charrois TL (2015). Systematic reviews: what do you need to know to get started?. Can J Hosp Pharm.

