# Supplementary figures and images for: Digital Characteristics and Dissemination Indicators to Optimize Delivery of Internet-Supported Mindfulness-Based Interventions for People With a Chronic Condition: Systematic Review
Source: JMIR Ment Health. 2018 Aug 21;5(3):e53. doi: 10.2196/mental.9645 (PMC6123540; doi:10.2196/mental.9645)

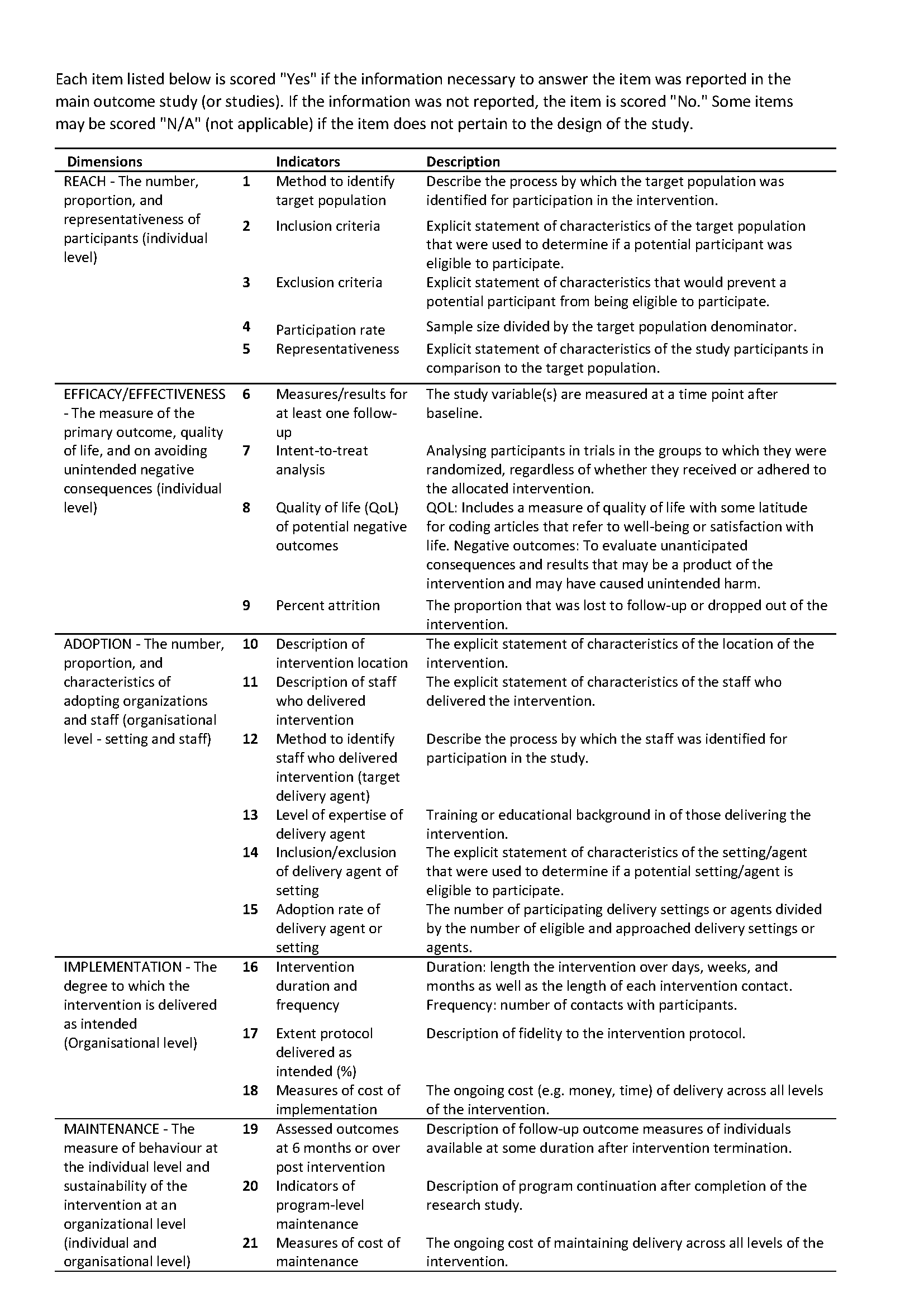

Supplement: Multimedia Appendix 1 [file mental_v5i3e53_app1.png]
